# NURSING HOME CULTURE CHANGE AND QUALITY OF LIFE AND HEALTH DEFICIENCIES: NATIONWIDE PANEL STUDY

**DOI:** 10.1093/geroni/igz038.240

**Published:** 2019-11-08

**Authors:** Michael J Lepore, Julie C Lima, Melissa A Clark, Pedro L Gozalo, Susan C Miller

**Affiliations:** 1 RTI International, Atlanta, Georgia, United States; 2 Brown University, Providence, Rhode Island, United States

## Abstract

Transforming nursing home (NH) cultures—from impersonal institutions to enriching communities where residents and employees thrive—requires multifaceted change. NHs deploy an array of culture change (CC) practices, in three core domains: resident-centered care, staff empowerment, and physical environment. This study uses novel data on CC practice from a nationally representative panel of NHs (N=1,585) surveyed in 2009/2010 and 2016/2017. To understand how changes in practice adoption may relate to quality changes we linked longitudinal data on the CC practice domain scores for resident-centered care, staff empowerment, and physical environment and on NH deficiencies relating to health and quality of life (QoL) and combined these with Certification and Survey Provider Enhanced Reporting baseline data. Multinomial logistic regressions incorporating survey weights and inverse probability of weight (to address CC selection) estimated the relative risk ratios (RRR) of increased CC practice corresponding with NHs having fewer or no change in deficiencies, versus increased deficiencies. NHs with much increase in staff empowerment scores had a higher likelihood of no change (compared to an increase) in health deficiencies (RRR 2.0; 95% CI 1.15, 3.61) and severe health deficiencies (RRR 2.03; 95% CI 1.05, 3.93). With a RRR of 1.63, NHs with any improvement in resident-centered care scores appeared to have a higher likelihood of no change (compared to an increase) in QoL deficiencies but statistical significance was not reached (p=0.11). This study provides some support for the benefits of CC practices, and in particular, supports the importance of staff empowerment practices in NHs.

